# A taxonomic review of the Gyrinidae (Coleoptera) in Korea

**DOI:** 10.3897/zookeys.509.9442

**Published:** 2015-06-24

**Authors:** Dae-Hyun Lee, Kee-Jeong Ahn

**Affiliations:** 1 Department of Biology, Chungnam National University, Daejeon 305-764, Republic of Korea

**Keywords:** Taxonomy, Gyrinidae, Coleoptera, Korea

## Abstract

A taxonomic review of Korean Gyrinidae is presented. Seven species [*Dineutus orientalis* (Modeer, 1776), *Gyrinus gestroi* Régimbart, 1883, *Gyrinus japonicus* Sharp, 1873, *Gyrinus pullatus* Zaitzev, 1908, *Orectochilus punctipennis* Sharp, 1884, *Orectochilus Regimbarti* Sharp, 1884 and *Orectochilus villosus* (Müller, 1776)] in three genera are recognized, one of which (*Orectochilus punctipennis* Sharp, 1884) is reported for the first time in Korea. We also found that *Gyrinus curtus* Motschulsky, 1866 previously recorded in Korea was an incorrect identification of *Gyrinus pullatus* Zaitzev, 1908. Habitus and SEM photographs, distribution maps, keys, and diagnoses of genera and species are provided.

## Introduction

The Gyrinidae are water beetles with unique swimming behavior where adults rapidly gyrate and whirl on the surface of water. They inhabit stagnant or slowly running water and prefer clean oxygen-rich habitats (Brinck 1955). Members of the Gyrinidae are characterized by the combination of the following characters: compound eyes divided completely with one pair on the dorsal surface of the head (above the water line) and another on the ventral surface of the head (below the water line); antenna short with a broad, cup-shaped scape, subtriangular pedicel, elongate but compact flagellum; middle and hind legs broadly expanded and fringed with setae for swimming (Miller and Bergsten 2012).

The whirligig beetles contain about 1000 species in 25 genera worldwide (Slipinski et al. 2011) and 118 species in seven genera in the Palaearctic region (Mazzoldi 2003). In Korea, seven species in three genera have been recorded (Jung et al. 2011), 50 species in six genera from China, 16 species in three genera from Japan and 11 species in three genera from the Far East of Russia (Mazzoldi 2003).

It was Kolbe (1886) who recorded the first gyrinid species, *Gyrinus japonicus* Sharp, 1873 in Korea. About 50 years later, Japanese entomologists, Takizawa (1931) and Kamiya (1936) reported two species (*Dineutus orientalis* Modeer, 1776 and *Gyrinus japonicus* Sharp, 1873) with descriptions and illustrations. Cho (1957) was the first Korean beetle taxonomist who studied Korean gyrinid fauna in detail. Since then, a few entomologists have studied Korean gyrinid beetles, mainly in the local fauna and no taxonomic review has been performed since Yoon (1988). Accordingly, this review is an updated contribution worth undertaking for Korean Gyrinidae.

In this paper we recognized seven species [*Dineutus orientalis* (Modeer, 1776), *Gyrinus gestroi* Régimbart, 1883, *Gyrinus japonicus* Sharp, 1873, *Gyrinus pullatus* Zaitzev, 1908, *Orectochilus punctipennis* Sharp, 1884, *Orectochilus Regimbarti* Sharp, 1884 and *Orectochilus villosus* (Müller, 1776)]: *Orectochilus punctipennis* Sharp is identified for the first time in Korea and *Gyrinus curtus* Motschulsky previously recorded in Korea was an incorrect identification of *Gyrinus pullatus* Zaitzev. We provided habitus and SEM photographs, distribution maps, keys, and diagnoses of genera and species.

## Materials and methods

To identify Korean Gyrinidae more reliably, we compared them with type and voucher specimens in the National History Museum (NHM, London, United Kingdom). The specimens used in this study are deposited in Chungnam National University Insect Collection (CNUIC), Daejeon, Korea and Ehime University Museum (EUMJ), Matsuyama, Japan. Habitus photographs were prepared from single or multi-layered shots taken with Olympus DP71 camera with several images amalgamated using Helicon Focus 5.3 (Helicon Soft Ltd, Kharkov, Ukraine) and edited by Adobe Photoshop CS4 (Adobe Systems, San Jose, CA, USA). Dry specimens for scanning electron microscope (SEM) photographs were sputter coated with platinum (Cressington 208 auto sputter coater, Hertfordshire, UK) and examined under SEM (S-4800, Hitachi, Tokyo, Japan). The terminology of taxonomic characters and measurements of specimens mainly follow Holmen (1987) and Miller and Bergsten (2012). The subdivision of China and Russia follows the standards of Löbl and Smetana (2003).

## Results

### Gyrinidae Latreille, 1810

#### Key to the genera of Korean Gyrinidae

**Table:** 

1	Pronotum with transverse depression on median part; elytra with rows of punctures	***Gyrinus***
–	Pronotum without transverse depression; elytra without rows of punctures	**2**
2	Labrum short and broad; elytra without compact setae	***Dineutus***
–	Labrum long and subtriangular; elytra with compact setae	***Orectochilus***

#### Genus Dineutus

Taxon classificationAnimaliaColeopteraGyrinidae

MacLeay, 1825

Figs 7–8 , 10–14

Dineutus MacLeay, 1825: 133. Type species: *Dineutus politus* MacLeay, 1825.

##### Diagnosis.

Head about 2.0 times as wide as long, with polygonal micro-reticulation. Clypealium with long setae. Antenna with 6 antennomeres. Galea absent; cardo and stipes with few setae on apico-lateral part. Pronotum (Fig. 7) convex without transverse groove; anterior margin bisinuate, posterior margin rounded, anterior angle acute, posterior angle nearly rectangular. Scutellum (Fig. 8) concealed when elytra closed. Elytra (Fig. 8) widest at middle, without punctato-striae and compact setae; subelytral suture absent. Prosternum (Fig. 10) transverse; anterior margin rounded; prosternal process (Fig. 10) linear-form and posterior margin rounded. Anterior margin of mesoventrite (Fig. 11) acute, posterior part bifid. Metaventrite (Fig. 12) broad, diamond-shaped, flattened; anterior part acute, lateral parts transverse. Sternite II (Fig. 13) without pit and groove. Sternite VIII (Fig. 14) with compact and short setae on lateral margins.

#### Subgenus Spinosodineutes

Taxon classificationAnimaliaColeopteraGyrinidae

Hatch, 1925: 447

Spinosodineutes Hatch, 1925: 447. Type species: *Gyrinus spinosus* Fabricius, 1781.
Gyrinodineutus Ochs, 1926: 66. Type species: *Dineutus unidentatus* Aubé, 1838. Synonymized by Brinck (1955: 104).

#### Dineutus (Spinosodineutes) orientalis

Taxon classificationAnimaliaColeopteraGyrinidae

(Modeer, 1776)

Figs 1 , 7–14 , 41–43 , 59

Gyrinus orientalis Modeer, 1776: 160.
Dineutus marginatus Sharp, 1873: 56. Synonymized by Ochs (1926: 136).
Dineutus quadrispina Fairmaire, 1878: 88. Synonymized by Ahlwarth (1910: 6).
Dineutus (Spinosodineutes) orientalis : Ochs (1930: 9).

##### Specimens examined.

**NORTH KOREA**: Gangwon Prov.: 1 ♂ 1♀, Uonsan-city, Anbyon-gun, Pisan-ri, 23.VII.2008, Changdo Han. **SOUTH KOREA**: Gangwon Prov.: 2 ♂♂ 1 ♀, Cheorwon-gun, Dongsong-eub, Odeok-ri, Hakji-reservoir, 15.IX.1990; Gyeongbuk Prov.: 3 ♂♂ 3 ♀♀, Euiseong-gun, Geumseo-myeon, Sujeong-ri, 27.VII.2010, SW Jung, DH Lee, valley (1 ♂, on slide); 1 ♂ 1 ♀, Gumi-si, Okgye-dong, 18.VI.1990, SH Lee; 1 ♀, Gyeongju-si, Geoncheon-eub, Sinpyeong-ri, 28.V.1993, SH Lee; 1 ♂, Gyeongju-si, Wolseong-dong, 5.VI.1987, HM Lee; Gyeonggi Prov.: 1 ♂ 2 ♀♀, Hwaseong-si, Songsan-myeon, Dokji-ri, N37°15'25.08", E126°40'49.68", 5 m, 4.VII.2013, DH Lee, SG Lee, pond near brackish zone; 1 ♀, Incheon-si, Ganghwa-gun, Gyodong-myeon, 6.X.2009, HM Lim; 1 ♂, same data as former except for, Naega-myeon, 16.IX.1990, SH Lee, pond; 1 ♀, Suwon-si, 24.VI.1969; Jeju Prov.: 1 ♂, Bukjeju-gun, Jocheon-eub, Seonheul-ri, 11.VI.2005, DH Lee, pond; 1 ♂, Seoguipo-si, Daejeong-eub, Boseong-ri, 9.VII.1985; Jeonnam Prov.: 1 ♂, Haenam-gun, Hwangsan-myeon, Namri-ri, 13.VI.2010, SH Lee; 1 ♂, Hawsun-gun, Dong-myeon, Jangdong-ri, 3.VIII.2009, SH Lee.

##### Published Korean records.

Dineutus (Spinosodineutes) orientalis: Mazzoldi (1995: 160); Mazzoldi (2003: 26). *Dineutus orientalis*: Kamiya (1936: 16); Mochizuki and Tsunekawa (1937: 78); Mochizuki and Matsui (1939: 56); Kamiya (1940: 130); Cho (1957: 201); Cho (1963: 46); Cho (1969: 191); Kim and Nam (1982: 25); Lee et al. (1985: 402); Kwon and Suh (1986: 98); Yoon (1988: 617); Lee et al. (1992: 55); Kim et al. (1994: 14); Lee (1995: 15); Hua (2002: 41); Han et al. (2007: 271); Han et al. (2008: 261); Park et al. (2008a: 248); Cho and Park (2010: 95). *Dineutus marinatus*: Okamoto (1924: 167); Takizawa (1931: 15); Yoshino (1935: 16); Kusanagi (1936: 323).

##### Diagnosis.

Length 9.0–10.0 mm. Dorsal surface mostly dark grey; clypeus, labrum, interorbital area metallic green; lateral parts of pronotum, margin of elytra yellow. Maxillary palpi shorter than labial palpi, palpomere 4 as long as 1–3 combined. Anterior margin of ligula slightly bisinuate; labial palpomere 3 as long as 1–2 combined. Gula with few setae on lateral margin. Postero-lateral and apical parts of elytron sharply pointed (arrows in Fig. 9). Antero-medial margin of prosternum (Fig. 10) strongly rounded. Prosternal process nearly parallel-sided (arrow in Fig. 10). Median lobe of aedeagus (Figs 41–43) shorter than paramere; apical part acute; sperm-groove as in Fig. 42. Paramere (Figs 41, 43) slightly curved at anterior fourth; apical margin rounded.

**Figures 1–6.**
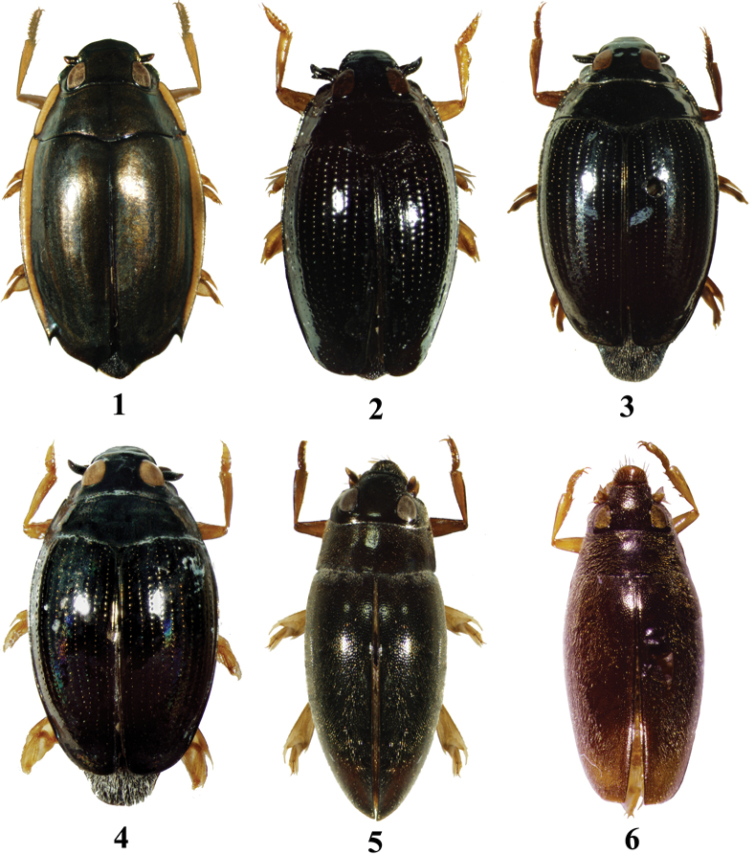
Habitus. **1** *Dineutus orientalis*, 9.0 mm **2** *Gyrinus gestroi*, 4.8 mm **3** *Gyrinus japonicus*, 7.0 mm **4** *Gyrinus pullatus*, 6.0 mm **5** *Orectochilus punctipennis*, 6.5 mm **6** *Orectochilus villosus*, 5.7 mm.

**Figures 7–14.**
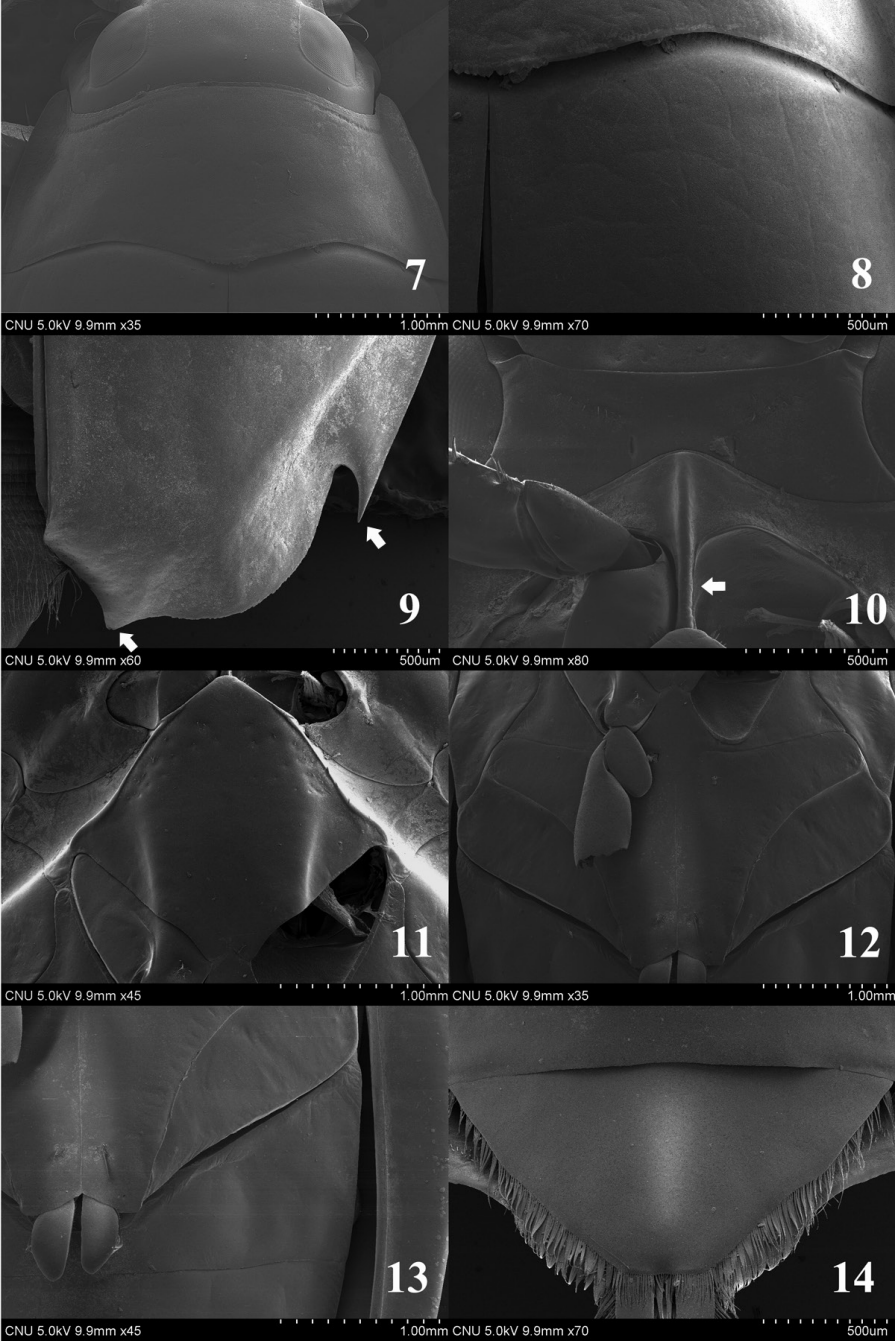
*Dineutus orientalis*. **7** pronotum (dorsal aspect) **8** basal part of elytron (dorsal aspect) **9** apical part of elytron (dorsal aspect) **10** prosternum (ventral aspect) **11** mesoventrite (ventral aspect) **12** metaventrite (ventral aspect) **13** sternite II (ventral aspect) **14** sternite VIII (ventral aspect).

##### Distribution.

Korea, China (Fujian, Guangdong, Guizhou, Guangxi, Hebei, Jiangsu, Liaoning), Japan, Russia (Far East) (Mazzoldi 2003).

##### Habitat.

Most Korean specimens are found in ponds or mountain streams with plentiful vegetation and low water temperature. Some specimens were collected in ponds near brackish water.

#### Genus Gyrinus

Taxon classificationAnimaliaColeopteraGyrinidae

Geoffroy, 1762

Figs 15–22

Gyrinus Geoffroy, 1762: 194. Type species: *Dytiscus natator* Linné, 1758.

##### Diagnosis.

**Figures 15–22.**
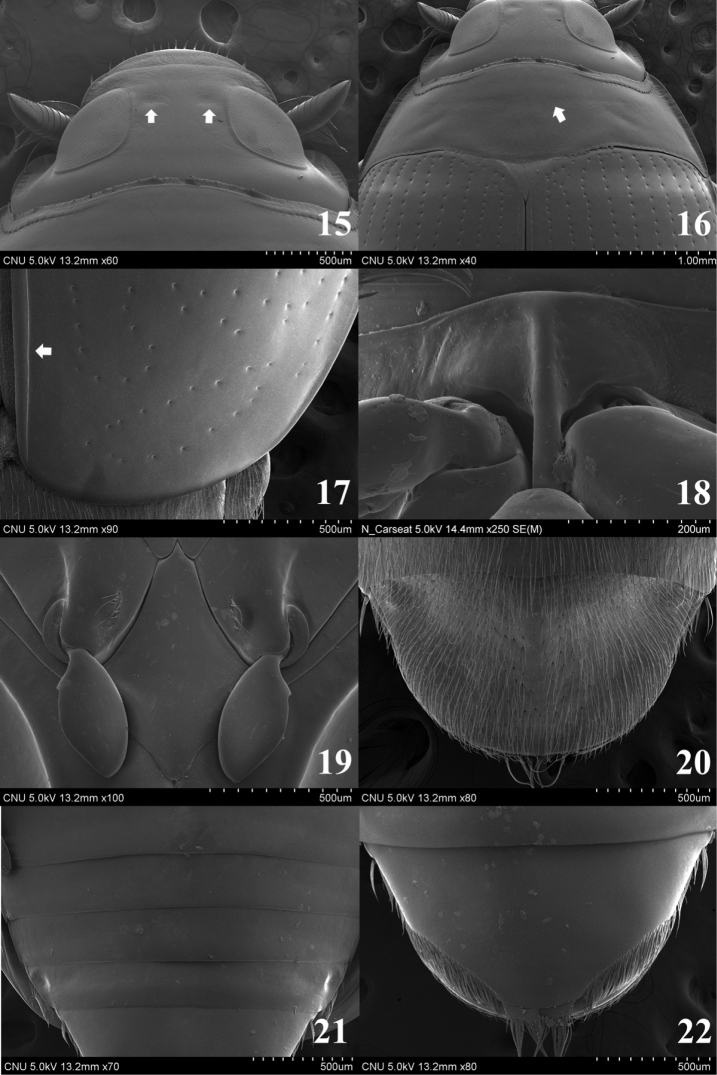
**15–17, 19–22** *Gyrinus japonicus* **18** *Gyrinus gestori* **15** head (dorsal aspect) **16** pronotum (dorsal aspect) **17** apical part of elytron (dorsal aspect) **18** prosternum (ventral aspect) **19** metaventrite (ventral aspect) **20** tergite VIII (dorsal aspect) **21** sternites IV–VII (ventral aspect) **22** sternite VIII (ventral aspect).

Head (Fig. 15) about 3.0 times wider than long, with micro-reticulation. Frons with two rounded depression (arrows in Fig. 15). Antenna with 9 antennomeres, antennomere 2 rugous with sparse punctures, 9 with compact short setae on apico-lateral part. Clypealium with long setae on antero-lateral part. Galea 1-articled. Pronotum with transverse depression on median parts (arrow in Fig. 16), lateral parts rugous, anterior margin slightly sinuate, posterior margin slightly rounded, anterior angle acute, posterior angle acute. Elytron (Fig. 16) with 11 punctato-striae and without compact setae, apical margin rounded; subelytral suture present (arrow in Fig. 17); epipleura reached on lateral part of sternite VII, anterior part of epipleura rounded. Prosternum transverse; anterior margin slightly rounded (Fig. 18). Prosternal process (Fig. 18) linear-form, gradually broad at posterior part. Metaventrite (Fig. 19) elongated, diamond-shaped, flattened; anterior part very acute; lateral parts curved upwardly. Tergite VIII (Fig. 20) with compact long setae and posterior margin rounded. Sternites III–VII (Fig. 21) with weak depression on lateral part. Sternite VII (Fig. 21) with long setae on lateral margin. Sternite VIII (Fig. 22) with long setae on postero-lateral parts and posterior margin rounded.

#### Subgenus Gyrinus

Taxon classificationAnimaliaColeopteraGyrinidae

Geoffroy, 1762

Gyrinus Geoffroy, 1762: 194. Type species: *Dytiscus natator* Linné, 1758.

##### Key to the species of Korean *Gyrinus*

**Table:** 

1	Hypomera and epipleura dark brown; apical part of median lobe less than 3.0 times as narrow as basal part	**2**
–	Hypomera and epipleura yellowish brown to brown; apical part of median lobe (Figs 50–52) more than 3.0 times as narrow as basal part	***Gyrinus pullatus***
2	Median part of mesoventrite (Fig. 23) with deep pit and large groove; median lobe (Figs 44–46) slightly shorter than paramere, apical margin rounded	***Gyrinus gestroi***
–	Median part of mesoventrite (Fig. 26) with shallow pit and vertical plica; median lobe (Figs 47–49) distinctly shorter than paramere, apical margin broadly rounded	***Gyrinus japonicus***

**Figures 23–31.**
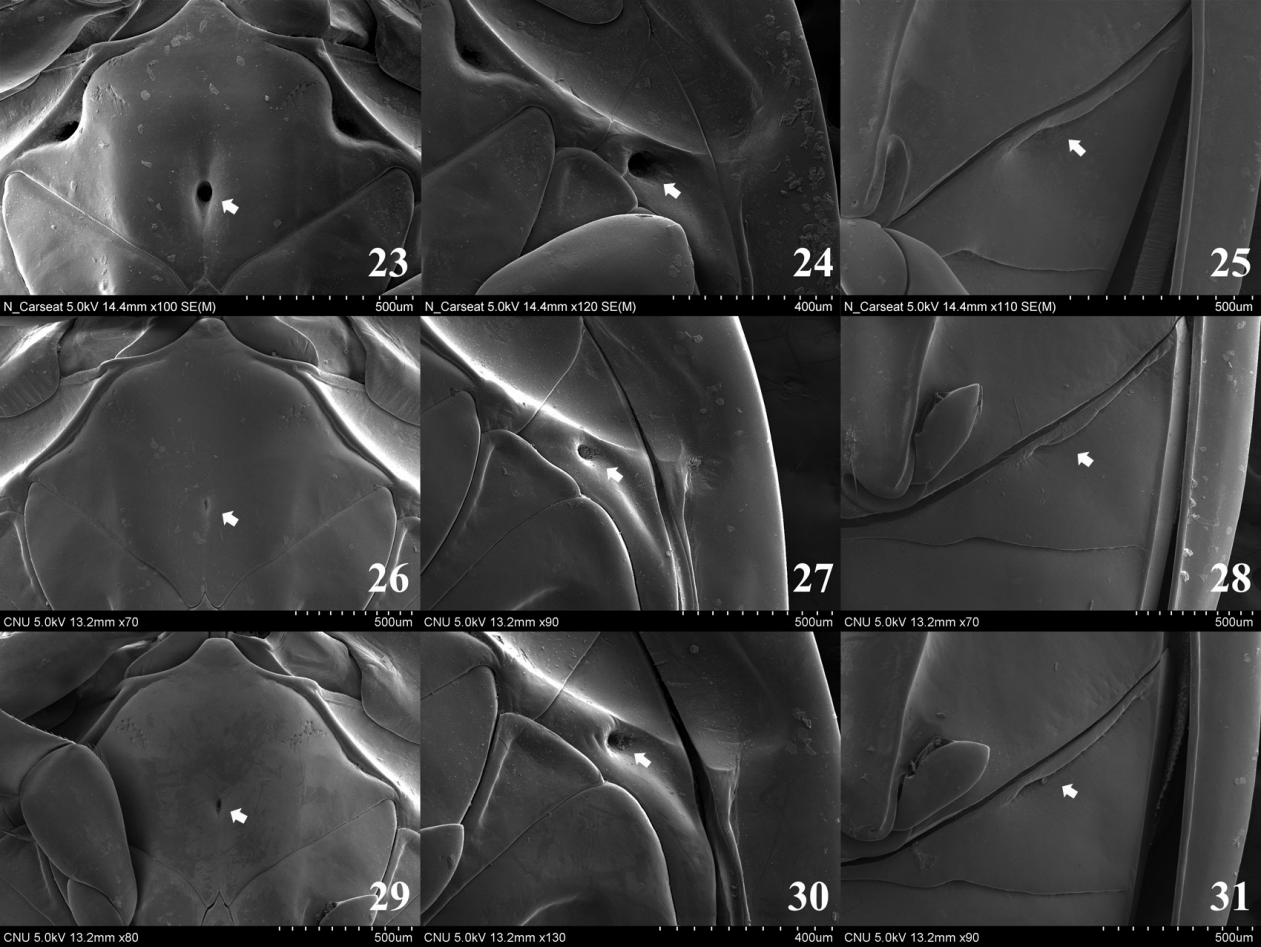
**23–25** *Gyrinus gestroi* **26–28** *Gyrinus japonicus* **29–31** *Gyrinus pullatus* **23** mesoventrite (ventral aspect) **24** metepisternum (ventral aspect) **25** sternite II (ventral aspect) **26** mesoventrite (ventral aspect) **27** metepisternum (ventral aspect) **28** sternite II (ventral aspect) **29** mesoventrite (ventral aspect) **30** metepisternum (ventral aspect) **31** sternite II (ventral aspect).

#### Gyrinus (s. str.) gestroi

Taxon classificationAnimaliaColeopteraGyrinidae

Régimbart, 1883

Figs 2 , 18 , 23–25 , 44–46 , 58

Gyrinus gestroi Régimbart, 1883: 165.

##### Specimens examined.

**SOUTH KOREA**: Jeju Prov.: 1 ♂, Bukjeju-gun, Jocheon-eub, Gyorae-ri, 10.V.1974; 1 ♂ 1 ♀, same data as former except for, 27.VII.2005, DH Lee, pond; 3 ♂♂ 3 ♀♀, same data as former except for, Seonheul-ri, 11.VI.2005 (1 ♂, on slide); 1 ♂, same data as former except for, 28.VII.2005, SH Lee, (1 ♂, on slide); 2 ♂♂ 1 ♀, same data as former except for, 22.V.2006; 2 ♂♂ 2 ♀♀, same data as former except for, 25.IX.2008; 1 ♂, same data as former except for, 15.VI.2011 (1 ♂, on slide); 2 ♂♂ 2 ♀♀, Jeju-si, Ara-dong, Gwaneum-temple, 22.VII.1990, SH Lee; 1 ♀, same data as former except for, Jeju National University, 11.VII.1985; 1 ♂, Seoguipo-si, Pyoseon-eub, 10.VII.1985; 1 ♀, Seoguipo-si, Seoho-dong, 23.VII.1990, SH Lee, pond; 1 ♂, Seoguipo-si, Seongsan-eub, Ojo-ri, 24.VII.1990, SH Lee.

##### Published Korean records.

*Gyrinus gestroi*: Kwon and Suh (1986: 98); Lee et al. (1992: 54); Kim et al. (1994: 134); Lee (1995: 13); Park et al. (2008: 247); Cho and Park (2010: 95).

##### Diagnosis.

Length 4.5–5.5 mm. Ventral surface mostly black; ventral part of antennomere 2, mouthparts, prosternum, legs, posterior part of sternite VII, sternite VIII yellowish brown to brown; hypomera, epipleura dark brown. Ligula with a row of sparse spines on antero-medial part. Anterior margin of mesoventrite rounded, posterior margin bifid with very deep pit (arrow in Fig. 23), large groove present on postero-medial and antero-lateral parts. Metepisternum with a deep pit (arrow in Fig. 24), oval groove present on anterior part. Sternite II with deep pit (arrow in Fig. 25), transverse groove on anterior part. Median lobe of aedeagus (Figs 44–46) nearly parallel-sided at middle to apex, slightly shorter than paramere; apical margin rounded; sperm-groove as in Fig. 45. Paramere (Figs 44, 46) broader than median lobe; apical margin broadly rounded.

##### Distribution.

Korea, Japan (Mazzoldi 2003).

##### Habitat.

All specimens were collected in ponds with plentiful vegetation in Jeju-do Island. They are frequently found together with *Dineutus orientalis* or *Gyrinus japonicus*.

#### Gyrinus (s. str.) japonicus

Taxon classificationAnimaliaColeopteraGyrinidae

Sharp, 1873

Figs 3 , 15–17 , 19–22 , 26–28 , 47–49 , 58

Gyrinus japonicus Sharp, 1873: 55.
Gyrinus (Gyrinus) japonicus : Cheo (1934: 210).
Gyrinus japonicus francki Zaitzev, 1953: 355. Synonymized by Mazzoldi (2003: 27).

##### Type specimens examined.

Syntypes: 1 ♂ 1 ♀ (NHM), with labels as follows: “*Gyrinus japonicus* Types D. S. Yokohama. Lewis, Type, Sharp Coll. 1905–313., Japan. G. Lewis.”.

##### Additional material examined.

**NORTH KOREA**: 6 ♂♂ 9 ♀♀, Pyongyang-city, Around Pyongyang-Hotel, Near Daedong-River, 15 vii 2008, Changdo Han; 16 ♂♂ 13 ♀♀, Pyongyang-city, Mt. Daesong-San, 24.VI.2009, Changdo Han. **SOUTH KOREA**: Chungbuk Prov.: 1 ♂, Yeongdong-gun, Yongsan-myeon, Sinhang-ri, 9.V.2009, SH Lee, pond; Chungnam Prov.: 1 ♂, Daejeon-si, Yusong-gu, Gyesan-dong, Sutonggol, 11.VII.1999, KJ Ahn, valley; 8 ♂♂ 12 ♀♀, Gongju-si, Banpo-myeon, Hakbong-ri, Mt. Gyeoryongsan, 25.VII.1992, SH Lee; 2 ♀♀, Yesan-gun, Deoksan-myeon, Sacheon-ri, Surak-temple, 26.VII.1995, SH Lee; Gangwon Prov.: 7 ♂♂ 6 ♀♀, Samcheok-si, Geundeok-myeon, Hamaengbang-ri, Chodang-reservoir, 7.IX.1990; Gyeongbuk Prov.: 1 ♀, Daegu-si, Buk-gu, Baekan-dong, 4.VI.1985; 13 ♂♂ 12 ♀♀, Gumi-si, Haepyeong-myeon, Songgok-ri, Dori-temple, 5.VIII.1994, SH Lee; 1 ♀, Gumi-si, Okgye-dong, 18.VI.1990, SH Lee; 1 ♂, Gunwi-gun, Hyoryeong-myeon, Gogok-ri, 6.VI.2010, SH Lee, pond; 4 ♂♂ 6 ♀♀, Gyeongju-si, Geoncheon-eub, Sinpyeong-ri, 28.V.1993, SH Lee; 1 ♀, Gyeongju-si, Jinhyeon-dong, Bulguk-temple, 23.VI.1991, SH Lee; 6 ♂♂ 6 ♀♀, Gyeongju-si, Naenam-myeon, Yongjang-ri, Hawgok-pond, 25.IV.1994, SH Lee, pond; 1 ♀, Kimcheon-si, Buhang-myeon, Daeya-ri, 29.VIII.2011, DH Lee, SW Jung, mountain stream; 36 ♂ 38 ♀♀, Pohang-si, Buk-gu, Jukjang-myeon, Duma-ri, 1.X.1989, SH Lee; 1 ♂ 2 ♀♀, Pohang-si, Gigye-myeon, Hwabong-ri, 5.IV.1993, SH Lee, pond; 1♂1♀, Sangju-si, Jungdong-myeon, Osang-ri, 18.VI.1990, SH Lee, pond; 1 ♂ 3 ♀♀, Uljin-gun, Seo-myeon, Wangpi-ri, 23.IV.1994, SH Lee, stream; 1 ♂, Ulsan-si, Ulju-gun, Samnam-myeon, Gacheon-ri 30.VI.2003, YB Cho, MJ Jeon, DH Lee, mountain stream (1 ♂, on slide); 8 ♂♂ 8 ♀♀, Yeongdeok-gun, Yeonghae-myeon, Myogok-ri, 6.VI.1994, SH Lee, pond; Gyeonggi Prov.: 1 ♂ 1 ♀, Anseong-si, 10.IX.1977, DW Oh; 1 ♀, Pocheon-si, Byeolnae-myeon, Yongam-ri, Mt. Yongamsan [= Sori-bong], 16.VII.1992, SH Lee; 1 ♀, Seoul-si, Gangbuk-gu, Ui-dong, Sogui-stream, 23.VI.2007, JG Lee, valley; 1 ♂, Yongin-si, Suji-gu, Sinbong-dong, 19.VII.1988, JH Lee; Gyeongnam Prov.: 1 ♂, Busan-si, Seo-gu, Ulmang-dong, 26.IV.2009, SH Lee; 1 ♂, Goseong-gun, Gaecheon-myeon, Bukpyeong-ri, 13.VIII.1995, SH Lee; 2 ♀♀, Euiryeong-gun, Yongdeok-myeon, Imok-ri, Deokam-pond, 18.V.2009, SH Lee, pond; 2 ♂♂ 5 ♀♀, Geoje-si, Geoje-myeon, Seosang-ri, Geoje-reservoir, 28.VII.2009, SH Lee, reservoir; 2 ♂♂, Habcheon-gun, Samga-myeon, Eojeon-ri, 8.V.2009, SH Lee, pond; 1 ♀, Hamyang-gun, Aneui-myeon, Sangwon-ri, Yongchu-temple, 16.VII.1985; 5 ♂♂ 2 ♀♀, Sacheon-si, Gonmyeong-myeon, Yongsan-ri, Dasol-temple, 14.VIII.1995, SH Lee; 1 ♂ 2 ♀♀, Sacheon-si, Sanan-myeon, 13.VIII.1995, SH Lee; 1 ♂, Sancheong-gun, Sancheong-eub, Jeonggok-ri, Jipum-church, 8.V.2009, SH Lee; Jeju Prov.: 1 ♂, Bukjeju-gun, Jocheon-eub, Gyorae-ri, 18.VIII.1992; 4 ♂♂ 3 ♀♀, Jeju-si, Ara-dong, Gwaneum-temple, 22.VII.1990, SH Lee (1 ♂, on slide); 1 ♂, Jeju-si, Hangyeong-myeon, Yongsu-ri, 17.VII.1992, SH Lee; Jeonbuk Prov.: 20 ♂♂ 27 ♀♀, Jeongeub-si, Naejang-dong, Mt. Naejangsan, 4.VIII.1990, SH Lee; 2 ♂♂ 2 ♀♀, Namwon-si, Sandong-myeon, Daesang-ri, Guijeong-temple, 28.VII.2008, DH Lee, pond; 6 ♂♂ 6 ♀♀, Namwon-si, Sannnae-myeon, Ibseok-ri, Silsang-temple, 12.VI.2008, DH Lee, pond (1 ♂, on slide); 1 ♀, Namwon-si, Unbong-eub, Maeyo-ri, 9.V.2009, SH Lee; Jeonnam Prov.: 2 ♂♂ 1♀, Haenam-gun, Songji-myeon, Geumgang-ri, 27.VII.2010, SH Lee, pond; 1 ♂, Gurye-gun, Gurye-eub, Sinseong-ri, Si-dong, 18.VI.2003, CH Park; 1 ♂, Janseong-gun, Bukha-myeon, Sinseong-ri, Mt. Naejangsan, 2.IV.2010, JC Jeong (1 ♂, on slide).

##### Published Korean records.

*Gyrinus japonicus*: Kolbe (1886: 179); Takizawa (1931: 18); Kamiya (1936: 20); Mochizuki and Tsunekawa (1937: 78) Mochizuki and Matsui (1939: 55); Ishii (1940: 43); Cho (1957: 201); Cho (1969: 191); Kim and Nam (1982: 25); Lee et al. (1985: 402); Yoon (1988: 616); Kim and Lee (1991: 65); Lee et al. (1992; 52); Lee (1994: 15); Lee (1995: 13); Mazzoldi (1995: 158); Nilsson et al. (2001: 29); Hua (2002: 41); Mazzoldi (2003: 27). *Gyrinus japonicus franki* (synonym): Kwon and Suh (1986: 98); Kim et al. (1994: 134); Kim (1995: 132); Han et al. (2007: 271); Han et al. (2008: 259); Cho and Park (2010: 95).

##### Diagnosis.

Length 6.5–8.5 mm. Ventral surface mostly black; mouthparts, prosternum, legs, sternite VIII brown to reddish brown; hypomera, epipleura dark brown. Ligula with a row of compact spines on antero-medial part. Anterior margin of mesoventrite acute and posterior margin bifid; shallow pit (arrow in Fig. 26) and vertical plica present on postero-medial part; shallow plica present on antero-lateral parts. Metepisternum with a pit (arrow in Fig. 27) and shallow groove on anterior part. Sternite II with small pit and transverse plica on anterior part (arrow in Fig. 28). Median lobe of aedeagus (Figs 47–49) parallel-sided at middle to apex, shorter than paramere, narrowest at middle; apical margin nearly straight; sperm-groove as in Fig. 48. Paramere as in Figs 47 and 49.

##### Distribution.

Korea, China (Northeast Territory), Japan, Russia (Far East) (Mazzoldi 2003).

##### Habitat.

Specimens were collected in ponds with plentiful vegetation and low water temperature. In summer, we often found that a large number of individuals gathered whirling on surface of water.

#### Gyrinus (s. str.) pullatus

Taxon classificationAnimaliaColeopteraGyrinidae

Zaitzev, 1908

Figs 4 , 29–31 , 44 , 46

Gyrinus pullatus Zaitzev, 1908: 244.

##### Specimens examined.

**SOUTH KOREA**: Gyeongbuk Prov.: 1 ♂, Gyeongju-si, Jinhyeon-dong, Bulguk-temple, 23.VI.1991, SH Lee; 1 ♂, Gyeongju-si, Naenam-myeon, Hwagok-ri, 25.IV.1993, SH Lee, pond; 1 ♂ 1 ♀, Pohang-si, Nam-gu, Yeongil-eub, 22.VI.1992, SH Lee; 1 ♀, Uljin-gun, Wonnam-myeon, Maehwa-ri, Maehwa-stream, 10.VI.1995, SH Lee; 1 ♀, Yeongdeok-gun, Changsu-myeon, Changsu-ri, 5.VI.1994, SH Lee, pond; 2 ♂♂, Yeongdeok-gun, Yeonghae-myeon, 17.VI.1985, SH Lee; 15 ♂♂ 15 ♀♀, Yeongdeok-gun, Yeonghae-myeon, Myogok-ri, 6.VI.1994, SH Lee, pond (1♂, on slide).

##### Published Korean records.

*Gyrinus pullatus*: Holmen (1987: 49); Mazzoldi (1995: 159); Nilsson et al. (2001: 31); Hua (2002: 41); Mazzoldi (2003: 28); *Gyrinus curtus* (misidentification): Mochizuki and Tsunekawa (1937: 78); Mochizuki and Matsui (1939: 56); Kim and Nam (1982: 25); Kwon and Suh (1986: 98); Kim et al. (1994: 134); Lee (1994: 15); Cho and Park (2010: 95).

##### Diagnosis.

Length 6.0–7.0 mm. Ventral surface mostly reddish brown; ventral part of antennomere 2, mouthparts, prosternum, legs, posterior part of sternite VII, sternite VIII yellowish brown to brown; hypomera, epipleura yellowish brown. Ligula with a row of sparse spines on antero-medial part. Anterior margin of mesoventrite rounded and posterior margin bifid; deep pit (arrow in Fig. 29) and vertical groove present on postero-medial part; deep groove present on antero-lateral parts. Metepisternum with a deep pit (arrow in Fig. 30) on anterior part. Sternite II with pit, transverse and thick plica on anterior part (arrow in Fig. 31). Median lobe of aedeagus (Figs 50–52) narrowed apically, shorter than paramere; narrowest at anterior fifth; apical margin nearly straight; sperm-groove as in Fig. 51. Paramere as in Figs 50 and 52.

##### Distribution.

Asia: Korea, China (Liaoning, Jilin), Russia (East Siberia, Far East), Europe: Estonia, Finland, Russia (North European Territory), Sweden (Mazzoldi 2003).

##### Remarks.

*Gyrinus curtus* was first recorded in Korea by Mochizuki and Tsunekawa (1937). After that, many entomologists [Mochizuki and Matsui (1939: 56); Kim and Nam (1982: 25); Kwon and Suh (1986: 98); Kim et al. (1994: 134); Lee (1994: 15); Cho and Park (2010: 95)] reported this species in Korea, only in the local fauna without any taxonomic comments. After examining specimens (2 ♂♂, Yeongdeok-gun, Yeonghae-myeon, 17.VI.1985, SH Lee) previously studied by Kwon and Suh (1986), Kim et al. (1994), Lee (1994), and Cho and Park (2010), we found that they had been incorrectly identified and actually represent *Gyrinus pullatus*. This species can be distinguished from *Gyrinus curtus* by the hypomera being yellowish brown to brown and the apical margin of the median lobe being nearly straight.

#### Genus Orectochilus

Taxon classificationAnimaliaColeopteraGyrinidae

Dejean, 1833

Figs 32–40

Orectochilus Dejean, 1833: 59. Type species: *Gyrinus villosus* Müller, 1776.

##### Diagnosis.

**Figures 32–40.**
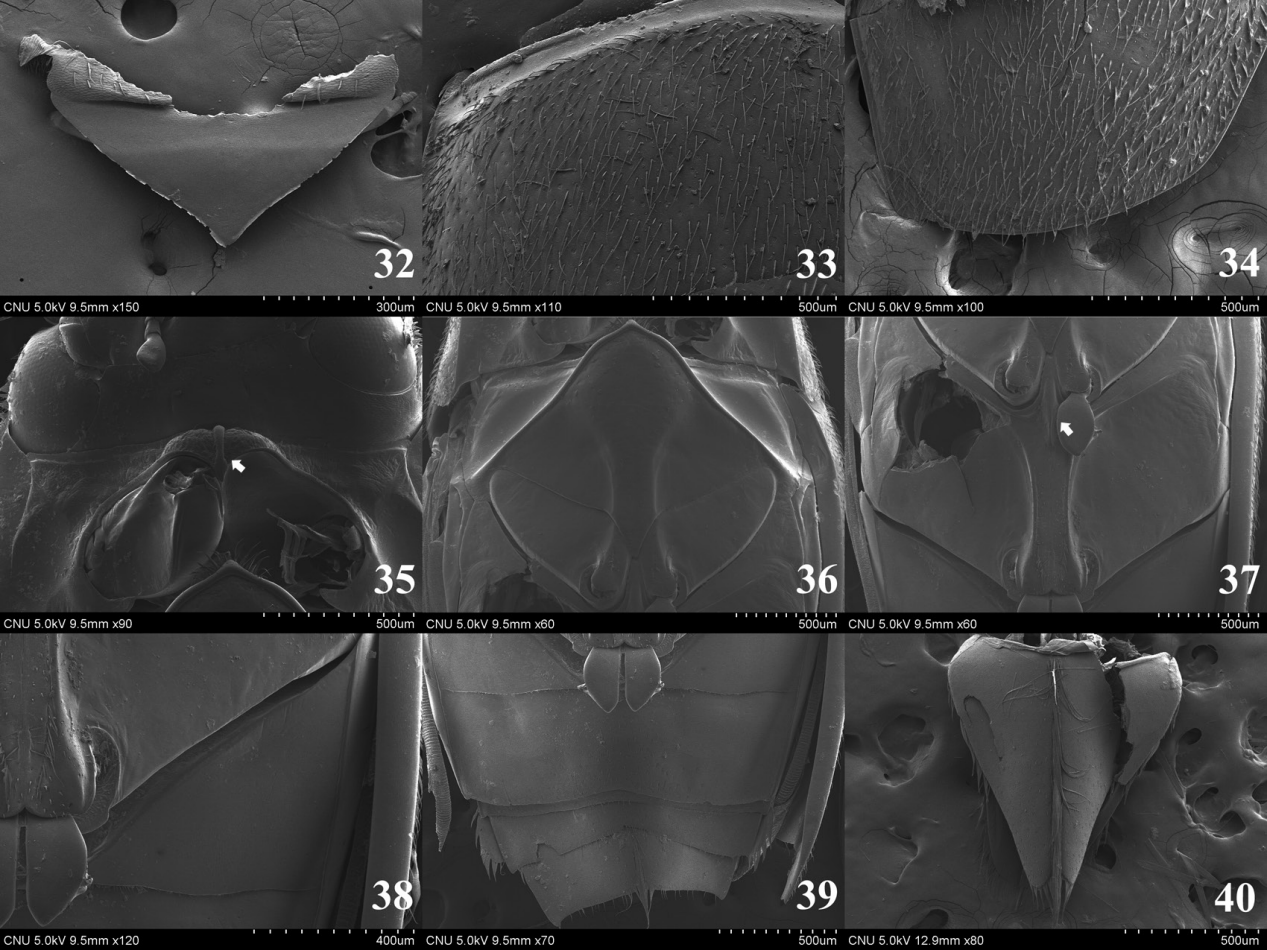
*Orectochilus villosus*. **32** scutellum (dorsal aspect) **33** basal part of elytron (dorsal aspect) **34** apical part of elytron (dorsal aspect) **35** prosternum (ventral aspect) **36** mesoventrite (ventral aspect) **37** metaventrite (ventral aspect) **38** sternite II (ventral aspect) **39** sternites III-VII (ventral aspect) **40** sternite VIII (ventral aspect).

Body long oval, with micro-reticulation, compact setae present on most dorsal part. Labrum semicircular, slightly wider than long, long setae present on anterior margin. Antenna with 9 antennomeres. Pronotum without transverse depression. Scutellum (Fig. 32) transverse and visible when elytra closed. Elytron (Figs 33, 34) with compact setae. Prosternum (Fig. 35) with few setae on anterior part. Prosternal process (arrow in Fig. 35) sagittiform, widest anterior three forth (Fig. 35). Mesoventrite (Fig. 36) with setae on anterior margin, vertical plica on postero-medial part; anterior part acute. Metaventrite cruciform, vertical process on median part (arrow in Fig. 37). Metepisternum (Fig. 36) without pit and groove. Metatibia with two spines of equal length. Sternite II (Fig. 38) without pit, transverse groove present on anterior part. Sternites IV–VI (Fig. 39) with short setae on median parts. Sternites VII–VIII (Figs 39, 40) with compact long setae on medial parts. Sternite VII (Fig. 39) with long setae on posterior margin. Sternite VIII (Fig. 40) longer than wide, long setae present on medial and lateral parts; apex bifid.

**Figures 41–55.**
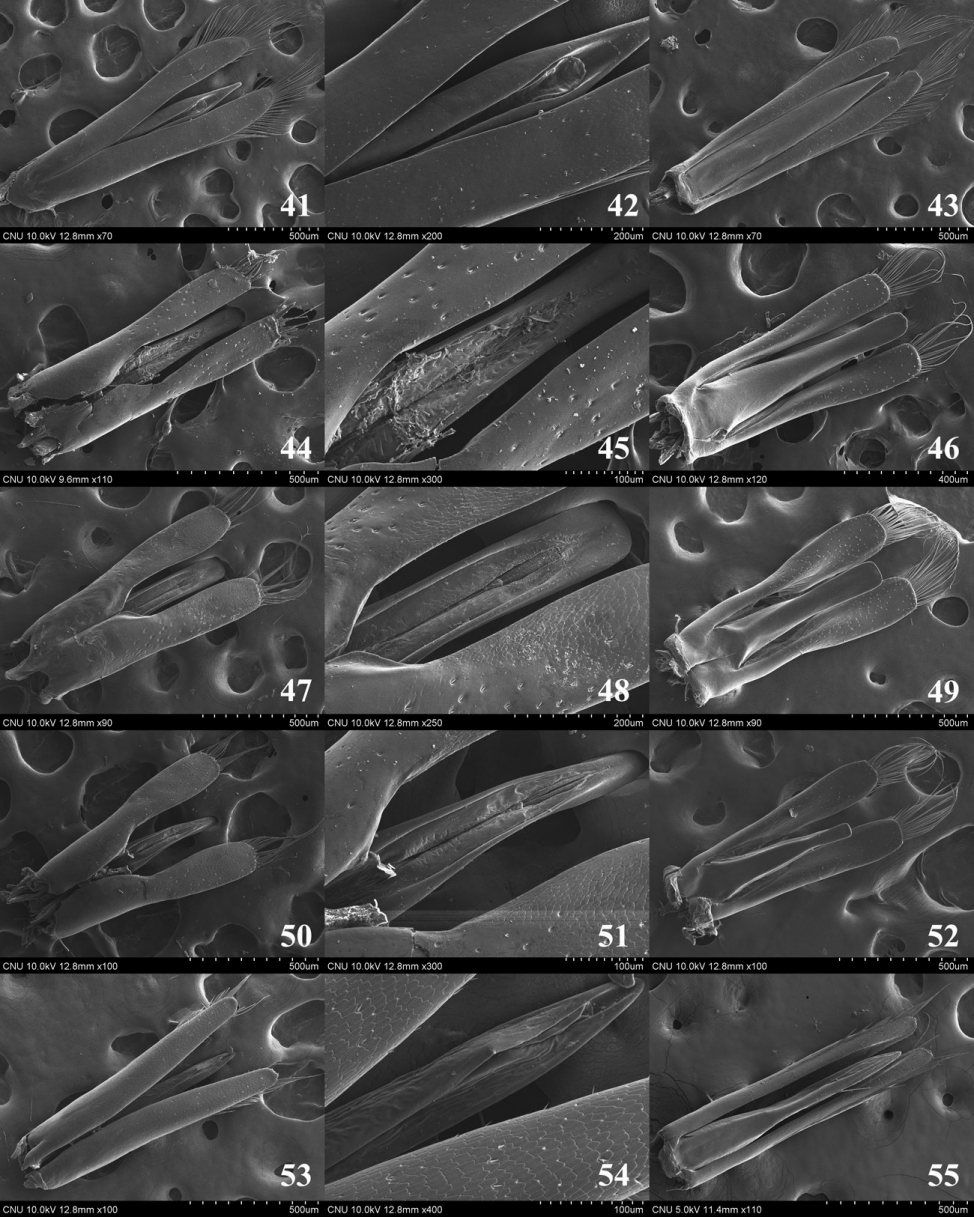
Aedeagus **41–43** *Dineutus orientalis* **44–46** *Gyrinus gestroi* **47–49** *Gyrinus japonicus* **50–52** *Gyrinus pullatus* **53–55** *Orectochilus villosus* **41** dorsal aspect **42** median lobe (dorsal aspect) **43** ventral aspect **44** dorsal aspect **45** median lobe (dorsal aspect) **46** ventral aspect **47** dorsal aspect **48** median lobe (dorsal aspect) **49** ventral aspect **50** dorsal aspect **51** median lobe (dorsal aspect) **52** ventral aspect **53** dorsal aspect **54** median lobe (dorsal aspect) **55** ventral aspect.

##### Key to the species of Korean *Orectochilus*

**Table:** 

1	Body less than 7.0 mm; apical part of elytron not protruded; median lobe of aedeagus shorter than paramere	**2**
–	Body more than 7.0 mm; apical part of elytron protruded; median lobe of aedeagus longer than paramere	***Orectochilus regimbarti***
2	Body black; apical part of elytra acute in dorsal view; paramere curved at middle, apical margin of gonocoxa (Fig. 56) rounded	***Orectochilus punctipennis***
–	Body brown; apical part of elytra broadly round in dorsal view; paramere nearly straight, apical margin of gonocoxa (Fig. 57) broadly rounded	***Orectochilus villosus***

**Figures 56–57.**
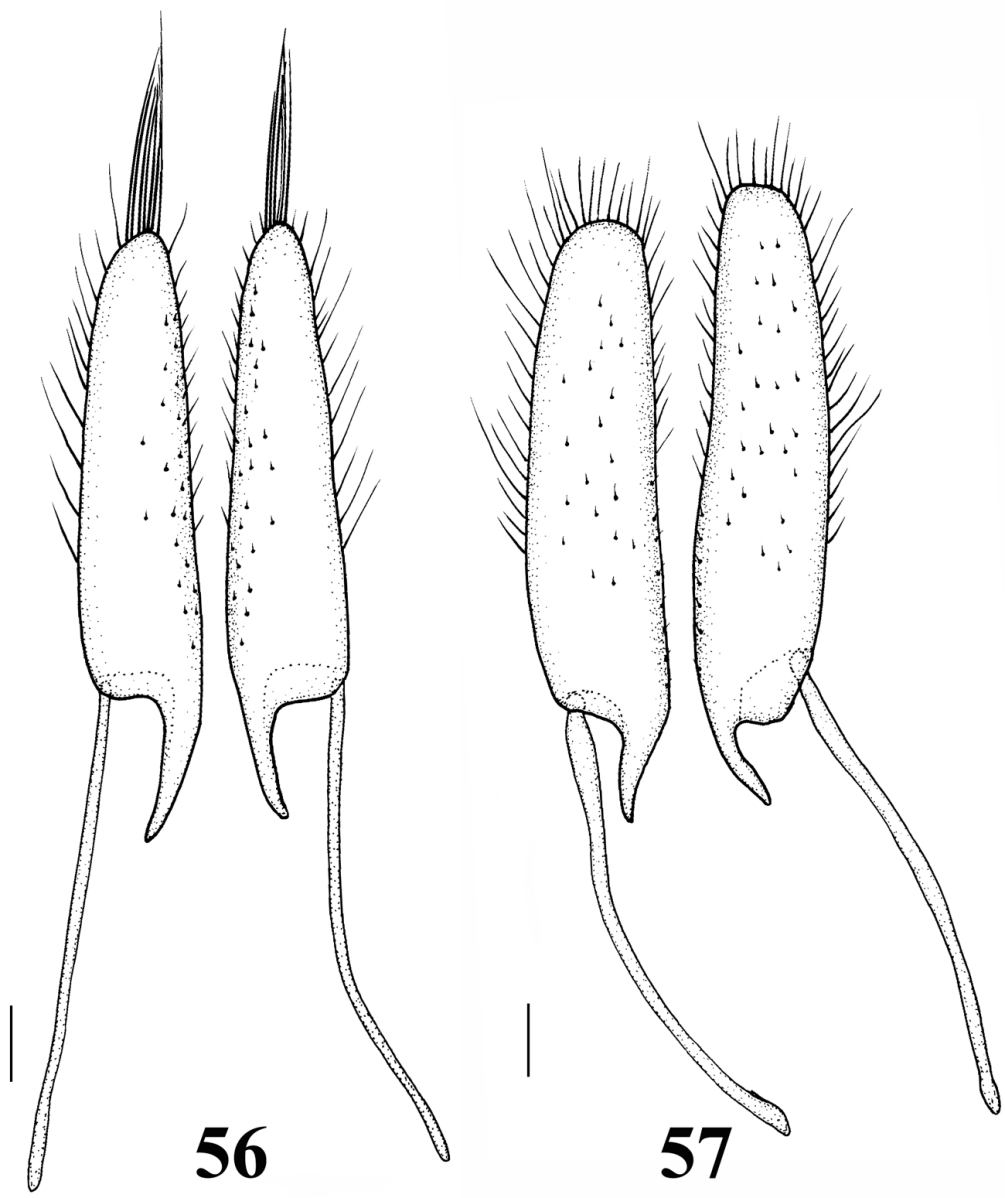
Gonocoxa. **56** *Orectochilus punctipennis* (dorsal aspect) **57** *Orectochilus villosus* (dorsal aspect). Scales = 0.1 mm.

#### Orectochilus punctipennis

Taxon classificationAnimaliaColeopteraGyrinidae

Sharp, 1884

Figs 5 , 56 , 59

Orectochilus punctipennis Sharp, 1884: 449.

##### Type material examined.

Syntype: 1 ♂ (NHM), with labels as follows: “Orectochilus punctipennis. Types D. S. Yokio. Japan. Lewis, Type, Japan. G. Lewis., Sharp Coll. 1905–313.”

##### Additional material examined.

**SOUTH KOREA**: Gangwon Prov.: 1 ♀, Gangneung-si, Okgyeo-myeon, Jusu-ri, 17.VIII.2011, SW Jung.

##### Diagnosis.

Length 6.5 mm. Dorsal and ventral surface mostly black; antenna, mouthparts, hypomera, epipleura, front leg brown; middle and hind legs, sternites V–VII yellowish brown. Head without pubescence on vertex and postero-lateral part. Pronotum widest at posterior margin; anterior angle rectangular; posterior angle rounded. Elytra (Fig. 5) widessst at middle; posterior margin acute in dorsal view. Protarsal claw as long as protarsomere 1. Posterior part of mesoventrite bifid. Median lobe of aedeagus shorter than paramere. Apical margin of gonocoxa (Fig. 56) rounded.

##### Distribution.

Korea, Japan, Russia (Far East) (Mazzoldi 2003).

##### Remarks.

*Orectochilus punctipennis* is recorded for first time in Korea. This species can be distinguished from *Orectochilus regimbarti* by the small size (less than 7.0 mm) and median lobe of aedeagus shorter than paramere. It also differs from *Orectochilus villosus* by the black body, posterior margin of the elytron acute, paramere curved at middle and apical margin of gonocoxa (Fig. 56) rounded.

##### Habitat.

A single female specimen was collected near the margin of a stream with plentiful vegetation and slow flow velocity.

**Figures 58–59.**
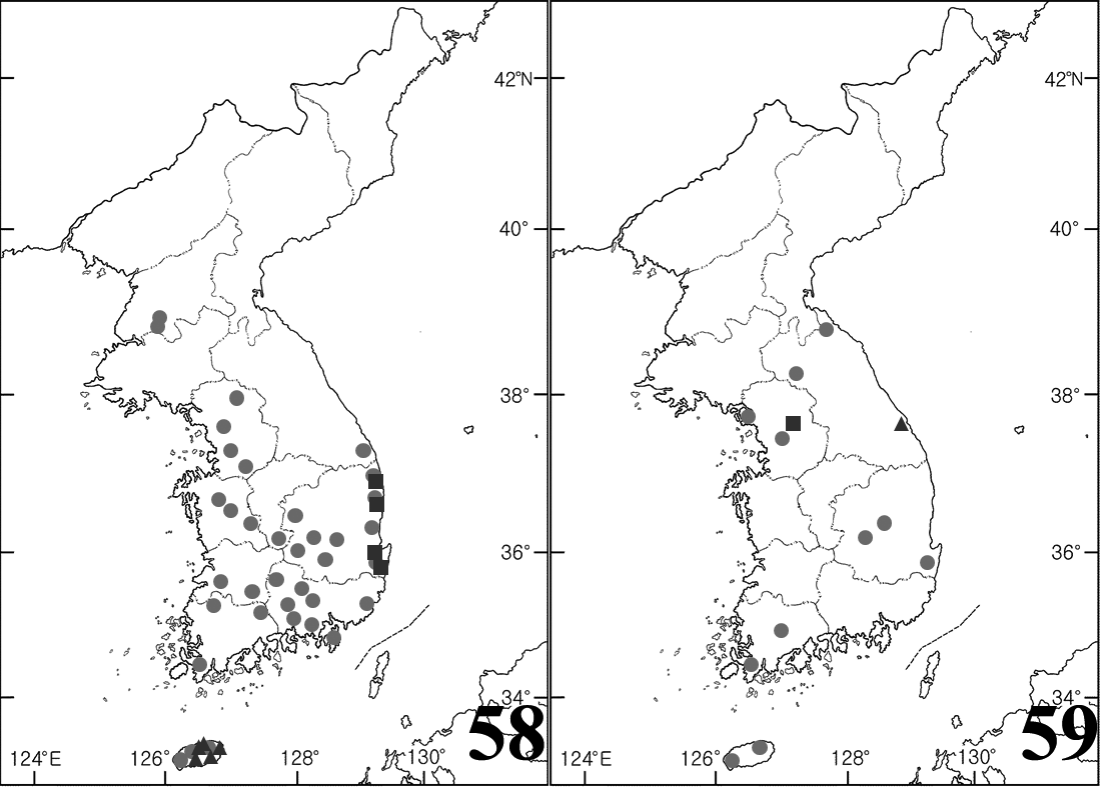
Distribution maps. **58** *Gyrinus japonicus* (circle), *Gyrinus gestroi* (triangle), *Gyrinus pullatus* (square) **59** *Dineutus orientalis* (circle), *Orectochilus punctipennis* (triangle), *Orectochilus villosus* (square).

#### Orectochilus regimbarti

Taxon classificationAnimaliaColeopteraGyrinidae

Sharp, 1884

Orectochilus regimbarti Sharp, 1884: 448.

##### Published Korean records.

*Orectochilus regimbarti*: Kwon and Suh (1986: 99); Kim et al. (1994: 134); Cho and Park (2010: 95).

##### Distribution.

Korea, Japan, Russia (Far East) (Mazzoldi 2003).

##### Remarks.

This species has been recorded in Korea by Kwon and Suh (1986), Kim et al. (1994) and Cho and Park (2010), only in their checklists without any taxonomic comments and specimens. We could not find any Korean specimens and the occurrence of this species in Korea is suspicious. However, its occurrence in Korea is possible because it is known from Japan and Russia (Far East).

#### Orectochilus villosus

Taxon classificationAnimaliaColeopteraGyrinidae

(Müller, 1776)

Figs 6 , 32–40 , 53–55 , 57 , 59

Gyrinus villosus Müller, 1776: 68.
Gyrinus modeeri Marsham, 1802: 100. Synonymized by Illiger (1807: 299).
Orectochilus villosus : Dejean 1883: 59.

##### Specimens examined.

**SOUTH KOREA**: Gyeonggi Prov.: 2 ♂♂ 3 ♀♀, Namyangju-si, Wabu-eub, Paldang-ri, Paldang-lake, 10.VI.1962, JS Lee (1 ♂ 1 ♀, on slide).

##### Published Korean records.

*Orectochilus villosus*: Yoon (1988: 615); Kim et al. (1994: 134); Cho and Park (2010: 96).

##### Diagnosis.

Length 5.5–6.5 mm. Dorsal surface dark brown; ventral surface mostly brown; ventral part of antennomere 2, mouthparts, hypomera, epipleura; legs yellowish brown. Head without pubescence on postero-lateral margins. Posterior angle of pronotum rectangular. Elytra (Fig. 34) widest at middle, posterior margin broadly rounded. Posterior part of mesoventrite (Fig. 36) slightly acute. Median lobe of aedeagus (Figs 53–55) slender, shorter than paramere; apical part acute; sperm-groove as in Fig. 54. Paramere (Figs 53, 55) nearly straight, long setae present on lateral and apical parts; apical part rounded. Apical margin of gonocoxa (Fig. 57) broadly rounded.

##### Distribution.

Europe, Asia; Korea, China (Liaoning), Cyprus, Iran, Iraq, Israel, Japan, Kazakhstan, Russia (East Siberia, Far East, West Siberia), Syria, Turkey, Uzbekistan (Mazzoldi 2003).

## Supplementary Material

XML Treatment for Dineutus

XML Treatment for Spinosodineutes

XML Treatment for Dineutus (Spinosodineutes) orientalis

XML Treatment for Gyrinus

XML Treatment for Gyrinus

XML Treatment for Gyrinus (s. str.) gestroi

XML Treatment for Gyrinus (s. str.) japonicus

XML Treatment for Gyrinus (s. str.) pullatus

XML Treatment for Orectochilus

XML Treatment for Orectochilus punctipennis

XML Treatment for Orectochilus regimbarti

XML Treatment for Orectochilus villosus
